# The trajectory of body mass index and blood pressure and fasting blood glucose in Chinese aged population: Cohort study

**DOI:** 10.1097/MD.0000000000047159

**Published:** 2026-01-09

**Authors:** Xinyu Zhao, Ying Jiang, Yun Li, Renying Xu

**Affiliations:** a Department of Preventive Medicine, School of Public Health, North China University of Science and Technology, Tangshan, Hebei, China; b Department of Clinical Nutrition, Ren Ji Hospital, Shanghai Jiao Tong University School of Medicine, Shanghai, China.

**Keywords:** aged population, blood pressure, body mass index (BMI), fasting blood glucose (FBG), trajectory

## Abstract

The purpose was to conduct a cohort study to investigate the association between body mass index (BMI) trajectory and future changes in blood pressure and fasting blood glucose (FBG) in Chinese aged people. This cohort included 2576 participants (1544 men and 1032 women; aged 67.7 ± 6.9 years) who were recruited in Shanghai, China. The trajectory of BMI was calculated based on BMI measurement in 2014, 2015 and 2016, and 3 BMI trajectories were determined: low-BMI (n = 418, 17.1%), medium-BMI (n = 1806, 68.5%) and high-BMI (n = 352, 14.4%). All participants were then followed up to 2022. The median follow-up time was 5 years. Systolic blood pressure (SBP), diastolic blood pressure (DBP), and FBG were annually measured. We used MIXED model to evaluate the relationship between BMI trajectory and changes in SBP, DBP and FBG during the follow up. The mean BMI for participants of low-, medium-, and high-BMI trajectories was 20.63 kg/m^2^, 24.52 kg/m^2^, and 28.80 kg/m^2^, respectively. BMI trajectory was significantly associated with future increase in blood pressure and FBG (*P* < .001). After adjustment of potential covariates, compared with low-BMI trajectory the mean increase in SBP was 4.9 mm Hg (95% CI: 2.5, 7.2 mm Hg) for those with medium-BMI trajectory and it was 3.8 mm Hg (95% CI: 0.7, 6.9 mm Hg) for those with high-BMI trajectory. It was 1.0 mm Hg (95% CI: −0.4, 2.4 mm Hg) and 2.2 mm Hg (95% CI : 0.4, 4.0 mm Hg) for DBP while it was 0.2 mmol/L (95% CI: 0, 0.4 mmol/L) and 0.3 mmol/L (95% CI: 0.1, 0.6 mmol/L) for FBG for those with medium-BMI and high-BMI trajectories compared with those low-BMI trajectory. Sex and age interacted with the association between BMI trajectory and changes in blood pressure and FBG. BMI trajectory was significantly associated with future increase in blood pressure and FBG in aged population, indicating maintenance of optimal BMI was important for the prevention of metabolic diseases.

## 1. Introduction

According to a Chinese report released in 2020, the prevalence of overweight in aged population increased from 31.9% in 2012 to 36.6% in 2018, and the prevalence of obesity rose from 11.6% to 13.6%. Obviously, overweight and obesity could increase blood pressure and fasting blood glucose (FBG), thus contribute to a high risk of developing hypertension and type 2 diabetes.^[1–3]^ From 2008 to 2018 in China, blood pressure in older and oldest adults (65–79 years and ≥80 years) increased by 0.8 to 3.3%, and the prevalence of hypertension increased by at least 8.2%,^[4]^ while the prevalence of hypertension in the elderly of Asia (65–74 years and ≥75 years) was 55.7% and 60.2% respectively in 2019.^[5]^ And people with high blood pressure were highly associated with the risk of death from cardiovascular disease.^[6,7]^ Similarly, diabetes also leaded to a huge burden on the old population, significantly increasing the risk of death from ischemic heart disease, stroke, chronic liver disease, tumors (liver cancer or breast cancer), and chronic genitourinary diseases in women. An estimation showed that 19.3% (≈135.6 million) of aged population (65–99 years) were confirmed with diabetes in 2019 while China, the United States and India ranked top 3 in countries with the highest number aged population with diabetes.^[8]^ The influencing factors of blood pressure and FBG changes in the aged population then should be deeply explored, to efficiently prevent hypertension, diabetes and cardiovascular diseases in the Chinese aged population.

Body mass index (BMI) is commonly used to assess weight status and predict the risk of chronic metabolic diseases, such as hypertension and diabetes.^[9]^ Previous studies have shown that BMI was an important factor associated with increasing blood pressure and FBG,^[10–12]^ but most previous studies focused on the BMI at a single point and ignored the dynamic change of BMI over time.^[13–15]^ Based on the conclusions of previous studies on BMI trajectory, both increasing BMI trajectory^[16,17]^ and high BMI trajectory^[18,19]^ lead to an increase in blood pressure and FBG in young or middle adults. The Nurses’ Health Study and the Health Professionals Follow-up Study found that participants with BMI trajectories from childhood through life was associated with the risk of type 2 diabetes, cardiovascular disease (CVD), and even death.^[20,21]^ However, some previous studies focused on children, adolescents, or young adults,^[22–25]^ few studies had analyzed the relationship in aged population.

In the current study, we calculated BMI trajectory using BMI data annually measured from 2014 to 2016 data in aged population recruited in Shanghai, China. We assumed that BMI trajectory might associate with future changes in blood pressure and FBG from 2017 to 2022.

## 2. Methods

### 2.1. Study population

All participants in the study were from communities in Shanghai, China. The initial number of adult participants were 3176 (1987 men and 1189 women), aged 60 or more and with 3 measurements of BMI during 2014 and 2016. We firstly excluded those with extremist values (≥99th percentile): systolic blood pressure (SBP) ≥ 220 mm Hg (n = 5), FBG ≥ 20 mmol/L or glycated hemoglobin ≥ 15% (n = 4), triglycerides ≥ 20 mmol/L (n = 4). Second, we excluded those whose follow-up visits were twice or less between 2017 and 2022 (n = 587, 18.5%). Finally, 2576 participants (1544 men and 1032 women) were included in the study (the details were shown in Fig. S1, Supplemental Digital Content, https://links.lww.com/MD/R131).

This study was a retrospective study and the ethics committee exempted informed consent. This study was approved by the Ethics Committee of Ren Ji Hospital, Shanghai Jiao Tong University School of Medicine (No. KY-2019-112).

### 2.2. Baseline data collection

The baseline survey was conducted in 2014, and we annually measured body weight (BW), height, blood pressure, FBG and other biochemical data from 2015 to 2016. Age and sex were abstracted from medical examination report. Height was annually measured with bare-foot by portable stadiometers by a weight measurement accuracy of 0.1 kg, and BW was annually measured in light cloth by calibrated beam scales with a height measurement accuracy of 0.5 cm. BMI is calculated by BW (kg) divided by the square of height (m). According to the cutoff criteria for adult BMI recommended by the Chinese Working Group on Obesity, BMI is divided into 3 categories: obese (BMI ≥ 28 kg/m^2^), overweight (24 kg/m^2^ ≤ BMI < 28 kg/m^2^) and underweight and normal (BMI < 24 kg/m^2^).^[26]^

Blood pressure was annually measured using an automatic blood pressure monitor (HBP-9020, Omron, China) after all participants rested for 10 minutes or more during the following period (2017–2022). If blood pressure was higher than normal, repeated measurement was further taken after another 10-minute rest and the average of the 2 measurements were recorded for further analysis.

After overnight fasting (≥8 hours), venous blood was drawn and transferred into a tube containing EDTA. FBG, serum total cholesterol (TC), triglycerides, alanine aminotransferase (ALT), aspartate aminotransferase (AST), and glycated hemoglobin A1c, were measured by enzyme-linked immunosorbent method. The estimated glomerular filtration rate (EGFR) was estimated by the Chronic Kidney Disease Epidemiological Collaboration (CKD-EPI) formula. Diabetes was defined as FBG ≥ 7 mmol/L. All the blood samples were analyzed in the Department of Laboratory Medicine, Ren Ji Hospital.

In the current study, changes in blood pressure and FBG during 2017 to 2022 were considered as the outcomes, which were calculated by mean blood pressure and FBG from 2017 to 2022 minus that from 2014 to 2016 each participant.

### 2.3. Follow up

All participants were annually followed up from 2017 to 2022. Blood pressure, FBG, TC, total triglycerides, ALT, AST and EGFR were annually measured by the same standards. A total number of 587 participants was excluded because they were followed <3 times. Participants in the study were lighter, with lower blood pressure and FBG than those out of the study (Table S1, Supplemental Digital Content, https://links.lww.com/MD/R131).

### 2.4. The calculation of BMI trajectory

**Figure 1.**
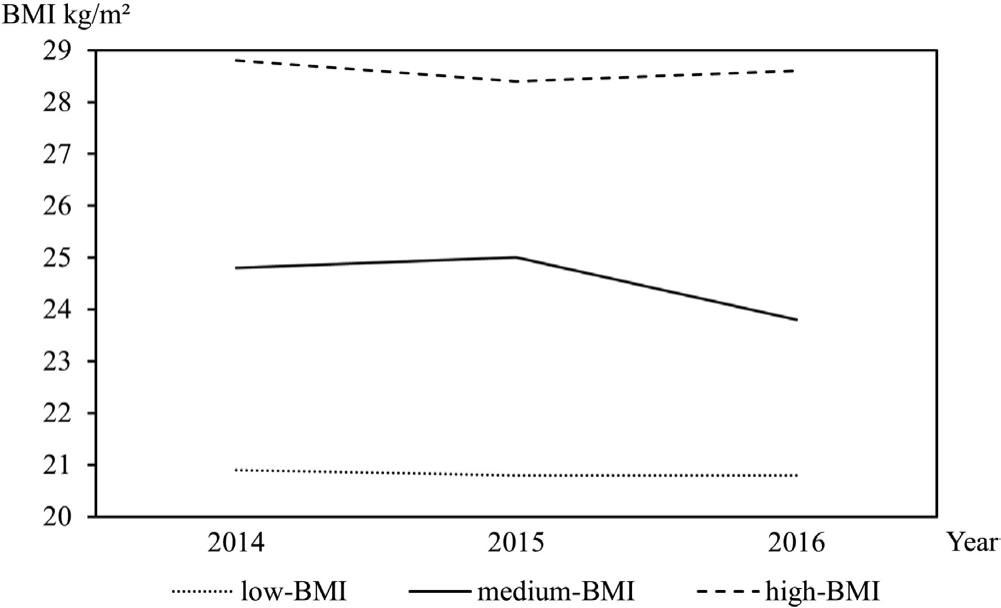
The trajectory of body mass index from 2014–2016 in 2576 Chinese aged participants. low-BMI medium-BMI high-BMI . BMI = body mass index

Based the group-based trajectory model, we used BMI data from 2014 to 2016 (Table S2, Supplemental Digital Content, https://links.lww.com/MD/R131) for trajectory analysis and divided all participants into 3 BMI trajectories: low (n = 418, 17.1%), medium (n = 1806, 68.5%), and high (n = 352, 14.4%) (Fig. 1). The criteria for selecting 3 trajectories were the mean BMI of each trajectory group in 3 years (Table S2, Supplemental Digital Content, https://links.lww.com/MD/R131).

### 2.5. Missing data

In our study, we had excluded participants who were followed up <3 times (n = 587). During the follow-up period from 2017 to 2022, we conducted statistics on the missing data of key variables. There were 62 to 342 (2.4–13.3%) and 64 to 345 (2.5–13.4%) participants with missing blood pressure and FBG annually. Multiple imputations were performed for all the above missing data for 5 times using FCS REG with SAS. The results with and without multiple imputations were similar (Table S3, Supplemental Digital Content, https://links.lww.com/MD/R131), so we reported using the original data.

### 2.6. Statistical analysis

All statistical analyses were conducted using SAS version 9.4 (SAS Institute, Inc., Cary). The formal hypothesis testing was 2-sided with a significant level of 0.05. Shapiro–Wilk test was used to test the normality of the continuous variables. If it was in normal distribution, data were presented as means and standard deviations, and if it was in abnormal distribution, data were presented as median and interquartile range, and analysis of variance was used to compare the differences in continuous variables between 3 BMI trajectories. The regression type for evaluating the relationship between trajectories and changes in blood pressure and FBG was linear regression. We used mixed-effects model to evaluate the relationship between BMI trajectories and changes in blood pressure (SBP and diastolic blood pressure (DBP)) and FBG from 2017 to 2022. The coefficients of the results were the mean difference in SBP, DBP, and FBG. This method allowed for examination at each time point, taking into account the interaction between blood pressure, FBG and BMI trajectory.

We included some potential covariates in 2 statistical models: model 1 was adjusted for age and sex; model 2 adjusted for age and sex, SBP (mm Hg), DBP (mm Hg), FBG (mmol/L), TC (mmol/L), triglycerides (mmol/L), ALT (U/L), AST (U/L), and EGFR (mL/min/1.73 m^2^).

We tested the interaction between BMI trajectories and age, sex, in the association with changes in SBP, DBP, and FBG. In order to test robustness, we conducted 3 sensitivity analysis: exclude participants with missing data, exclude participants with history of hypertension, and exclude patients with history of diabetes. Due to the influence of age and sex on the association between BMI trajectory and future changes in blood pressure and FBG, we conducted interaction tests and subgroup analysis on these 2 variables, and adjusted for the above covariates in model 2.

## 3. Results

The study was followed up for 6 years. In the current study, the mean age was 69.7 ± 6.7 years, the mean BMI was 23.9 ± 3.0 kg/m^2^, the mean systolic and DBP was 139.8 ± 17.2 mm Hg and 76 ± 10.3 mm Hg, respectively while the mean FBG was 5.7 ± 1.1 mmol/L in 2016. There were significant differences in age, height, BW, BMI, blood pressure, FBG and other biochemical data among 3 BMI trajectories (Table 1). The changes in blood pressure and FBG in 3 BMI trajectory were shown at Figures S2–S4, Supplemental Digital Content, https://links.lww.com/MD/R131.

**Table 1:** Basic characteristics of 2576 Chinese aged participants between 3 BMI trajectories in 2016.

	Low-BMI (n = 418)	Medium-BMI (n = 1806)	High-BMI (n = 352)	*P* value
Age, yr	71.2 ± 8.0	69.4 ± 6.3	69.6 ± 6.6	<.001
Height, cm	161.3 ± 8.0	161.6 ± 8.1	162.9 ± 8.3	.02
BW, kg	53.8 ± 6.5	62.4 ± 9.5	76.6 ± 9.1	<.001
BMI, kg/m^2^	20.6 ± 1.6	23.7 ± 2.0	28.8 ± 2.2	<.001
SBP, mm Hg	135.9 ± 18.9	139.5 ± 16.1	145.6 ± 18.7	<.001
DBP, mm Hg	74.5 ± 10.9	75.7 ± 9.8	79.6 ± 11.2	<.001
FBG, mmol/L	5.4 ± 1.1	5.7 ± 1.1	6.0 ± 1.4	<.001
TC, mmol/L	5.1 ± 1.0	5.4 ± 1.2	4.9 ± 1.1	<.001
TG, mmol/L	1.1 (0.8, 1.6)	1.7 (1.1, 1.8)	1.6 (1.2, 2.3)	<.001
ALT, U/L	15.5 (12.0, 20.0)	15.0 (14.0, 21.0)	21.0 (15.9, 29.3)	<.001
AST, U/L	21.5 ± 7.4	19.6 ± 7.6	23.4 ± 9.8	<.001
EGFR, mL/min/1.73 m^2^	77.9 ± 17.8	80.9 ± 19.4	91.3 ± 21.7	<.001
HbA1c, %	5.8 ± 0.6	6.0 ± 0.7	6.2 ± 0.9	<.001

ALT = alanine aminotransferase, AST = aspartate aminotransferase, BMI = body mass index, BW = body weight, DBP = diastolic blood pressure, EGFR = estimated glomerular filtration rate, FBG = fasting blood glucose, HbA1c = glycosylated hemoglobin, type A1C, SBP = systolic blood pressure, TC = total cholesterol, TG = total triglycerides.

Three BMI trajectories were determined based on BMI measured from 2014 to 2016: low-BMI (n = 418, 17.1%), medium-BMI (n = 1806, 68.5%), and high-BMI (n = 352, 14.4%) (Fig. 1). BMI remained stable within each BMI trajectory. BMI trajectory was associated with significant increase in future blood pressure and FBG during follow up (*P* < .001). After adjustment of potential covariates, the mean increase in SBP was 4.9 mm Hg (95% CI: 2.5–7.2 mm Hg) between those with medium-BMI and low-BMI trajectory and it was 3.8 mm Hg (95% CI: 0.7–6.9 mm Hg) between high-BMI and low-BMI trajectory. It was 1.0 mm Hg (95% CI : −0.4–2.4 mm Hg) and 2.2 mm Hg (95% CI: 0.4–4.0 mm Hg) for DBP while it was 0.2 mmol/L (95% CI : 0–0.4 mmol/L) and 0.3 mmol/L (95% CI: 0.1–0.6 mmol/L) for FBG for those with medium-BMI and high-BMI trajectory compared with those low-BMI trajectory (Table 2, Model 2). Obviously, the association between BMI trajectory and SBP and FBG was stronger in high-BMI than that in medium-BMI trajectory (Table 2).

**Table 2:** The mean difference and 95% CI for the association between BMI trajectory and future change in blood pressure and FBG in 2576 Chinese aged population.

	Low-BMI (n = 418)	Medium-BMI (n = 1806)	High-BMI (n = 352)	*P* value
SBP				
Model 1	0 (ref)	8.1 (5.6, 10.6)	9.5 (6.3, 12.7)	<.001
Model 2	0 (ref)	4.9 (2.5, 7.2)	3.8 (0.7, 6.9)	<.001
DBP				
Model 1	0 (ref)	2.1 (0.7, 3.6)	4.3 (2.5, 6.2)	<.001
Model 2	0 (ref)	1.0 (−0.4, 2.4)	2.2 (0.4, 4.0)	<.001
FBG				
Model 1	0 (ref)	0.4 (0.2, 0.6)	0.7 (0.4, 0.9)	<.001
Model 2	0 (ref)	0.2 (0.01, 0.4)	0.3 (0.1, 0.6)	<.001

Model 1: Adjusted for age (years) and sex; Model 2: Adjusted for variables in model 1 and further adjusted by systolic blood pressure (mm Hg), diastolic blood pressure (mm Hg), fasting blood glucose (mmol/L), total cholesterol (mmol/L), total triglycerides (mmol/L), alanine aminotransferase (U/L), aspartate aminotransferase (U/L) and estimated glomerular filtration rate (mL/min/1.73 m^2^). If the analysis was performed in subgroup, the related variable was not included in the model (e.g., SBP was not included in the model when the analysis was performed with SBP).

BMI = body mass index, CI = confidence interval, DBP = diastolic blood pressure, FBG = fasting blood glucose, SBP = systolic blood pressure.

The association between BMI trajectory and increase in blood pressure and FBG was interacted by sex and age (*P* < .001) (Table 3). In subgroup analysis, the association between BMI trajectory and blood pressure changes was more pronounced in men and younger old, while the association with FBG changes was more pronounced in women and older adults, relative to their counterparts (Table 3).

**Table 3:** The mean difference and 95% CI for the association between BMI trajectory and future change in blood pressure and FBG in 2576 Chinese aged participants: stratified by sex and age.

			Low-BMI	Medium-BMI	High-BMI	*P*-value
Sex	Men (n = 1544)	SBP	0 (ref)	6.5 (3.2, 9.8)	4.3 (0.1, 8.4)	<.001
		DBP	0 (ref)	2.2 (0.2, 4.2)	2.9 (0.4, 5.4)	<.001
		FBG	0 (ref)	0.1 (−0.2, 0.4)	0.3 (−0.1, 0.6)	<.001
	Women (n = 1032)	SBP	0 (ref)	3.1 (−0.3, 6.5)	4.1 (−0.7, 8.8)	<.001
		DBP	0 (ref)	−0.6 (−2.6, 1.4)	1.3 (−1.5, 4.1)	<.001
		FBG	0 (ref)	0.3 (0, 0.6)	0.3 (−0.1, 0.6)	<.001
Age	<70 yr (n = 1724)	SBP	0 (ref)	5.1 (1.9, 8.3)	4.4 (0.3, 8.5)	<.001
		DBP	0 (ref)	1 (−0.9, 2.8)	2.2 (−0.2, 4.6)	<.001
		FBG	0 (ref)	0.1 (−0.2, 0.4)	0.3 (0, 0.7)	<.001
	≥70 yr (n = 852)	SBP	0 (ref)	4.5 (1.0, 8.0)	4 (−0.8, 8.7)	<.001
		DBP	0 (ref)	1.3 (−0.8, 3.5)	2.2 (−0.7, 5.1)	<.001
		FBG	0 (ref)	0.3 (0, 0.6)	0.3 (−0.1, 0.6)	<.001

Model was adjusted for age (years), sex, systolic blood pressure (mm Hg), diastolic blood pressure (mm Hg), fasting blood glucose (mmol/L), total cholesterol (mmol/L), total triglycerides (mmol/L), alanine aminotransferase (U/L), aspartate aminotransferase (U/L) and estimated glomerular filtration rate (mL/min). Results were based on model 2; If the analysis was performed in subgroup, the related variable was not included in the model (e.g., SBP was not included in the model when the analysis was performed with SBP).

BMI = body mass index, CI = confidence interval, DBP = diastolic blood pressure, FBG = fasting blood glucose, SBP = systolic blood pressure.

We tested the interaction effects between BMI trajectories and age, sex, and found that except for the interaction between age and trajectories for changes in SBP, there was no significant interaction between trajectories and age and sex for changes in SBP, DBP and FBG (*P* > .05). The mean increase of the interaction between BMI trajectories and age for changes in SBP was 0.72 mm Hg (95% CI: 0.59–0.86 mm Hg) between those with medium-BMI and low-BMI trajectory and it was 0.57 mm Hg (95% CI: 0.31–0.82 mm Hg) between high-BMI and low-BMI trajectory (*P* < .001).

In the sensitivity analysis, we further studied the correlation between BMI trajectory and future changes in blood pressure and FBG. After excluding participants with missing data (n = 1302), history of hypertension (n = 1701), or history of diabetes (n = 443), some results are different from the main analysis (Table 4). We found that after excluding participants with missing data, the association with medium-BMI trajectory for DBP and FBG was not significant. After excluding participants with history of hypertension, the association with medium-BMI and high-BMI trajectories for DBP and FBG was not significant. After excluding participants with history of diabetes, those with high-BMI for FBG was not significant.

**Table 4:** The mean difference and 95% CI for the association between BMI trajectory and future change in blood pressure and FBG in 2576 Chinese aged participants: sensitivity analysis.

	Low-BMI (n = 418)	Medium-BMI (n = 1806)	High-BMI (n = 352)	*P*-value
Sensitivity-1				
SBP	0 (ref)	5.4 (1.8, 9.0)	4.7 (−0.2, 9.7)	<.001
DBP	0 (ref)	0.7 (−1.4, 2.8)	3.4 (0.5, 6.3)	<.001
FBG	0 (ref)	0.1 (−0.2, 0.3)	0.3 (−0.1, 0.7)	<.001
Sensitivity-2				
SBP	0 (ref)	5.1 (1.9, 8.3)	6.8 (1.4, 12.1)	<.001
DBP	0 (ref)	0.2 (−1.9, 2.2)	2.6 (−0.8, 6.0)	<.001
FBG	0 (ref)	0.1 (−0.2, 0.4)	0.3 (−0.2, 0.8)	<.001
Sensitivity-3				
SBP	0 (ref)	4.9 (2.4, 7.3)	3.6 (0.2, 6.9)	<.001
DBP	0 (ref)	1.1 (−0.3, 2.6)	2.4 (0.4, 4.4)	<.001
FBG	0 (ref)	0.1 (0, 0.2)	0 (−0.1, 0.2)	<.001

Sensitivity-1: Exclude participants with missing blood pressure (n = 1302); Sensitivity-2: Exclude participants with hypertension (n = 1701); Sensitivity-3: Exclude participants with diabetes (n = 443); Model was adjusted for age (years), sex, systolic blood pressure (mm Hg), diastolic blood pressure (mm Hg), fasting blood glucose (mmol/L), total cholesterol (mmol/L), total triglycerides (mmol/L), alanine aminotransferase (U/L), aspartate aminotransferase (U/L) and estimated glomerular filtration rate (mL/min/1.73 m^2^). Results were based on model 2. If the analysis was performed in subgroup, the related variable was not included in the model (e.g., SBP was not included in the model when the analysis was performed with SBP).

BMI = body mass index, CI = confidence interval, DBP = diastolic blood pressure, FBG = fasting blood glucose, SBP = systolic blood pressure.

## 4. Discussion

In this cohort study of 2576 aged population (≥60 years), we found that compared with low-BMI trajectory, the association between high-BMI trajectory and SBP, DBP, and FBG was significantly stronger than that of medium-BMI trajectory. And the association of BMI trajectory with blood pressure was more significant in men and younger old, while that with FBG was more obvious in women and older adults. Help doctors accurately assess hypertension and diabetes risks via BMI trajectories, create customized interventions for different population, highlight the value of early BMI trajectory identification and management, prevent diseases, and cut medical costs.

Some previous studies reported different BMI trajectories. Drøyvold et al^[27]^ found that an increase-BMI trajectory was significantly associated with increase in SBP and DBP, compared to a stable-BMI trajectory in both men and women and in all age groups, especially among those who were 50 years and older. However, this study evaluated the relationship between BMI trajectory and blood pressure through 2 cross-sectional surveys, and could not reflect the dynamic changes of BMI trajectory. One study evaluated the relationship between BMI trajectory throughout childhood and subsequent changes in blood pressure and found that compared with participants in the “lean-stable-increase” group, those in the “medium-marked-increase” (16.3 ± 1.5–21.3 ± 1.7 kg/m^2^) and “heavy-marked increase”(19.6 ± 2.9–26.4 ± 3.2 kg/m^2^) groups were more likely to have high blood pressure.^[28]^ However, the study population were children (aged 3–13 years). Gao et al^[29]^ evaluated the association of BMI trajectory and the outcomes of hypertension among 13,263 participants (≥35 years) in rural areas of China and found that BMI trajectory was significantly associated with the incidence of hypertension, when BMI increased during a median follow-up period of 4.8 years, the risks of hypertension were 1.103 (95% CI: 1.068–1.139) for those whose baseline BMI was within normal range (18.5–24.0 kg/m^2^). In our study, stable medium-BMI group (n = 1806, 68.5%) accounted for the majority, in which BMI remained at a low level within overweight range. The average BMI was at nearly 21.0 kg/m^2^ and 29.0 kg/m^2^ for participants with low-BMI and high-BMI trajectory. Only one study performed in the United States identified 3 similar BMI trajectories: normal weight (BMI ~24 kg/m^2^, 27.6%), overweight (BMI ~26 kg/m^2^, 65.1%), and obesity (BMI ~31 kg/m^2^, 7.3%) in 3861 participants (65–105 years).^[30]^ Our results showed that both medium-BMI and high-BMI trajectory had significant effects on future blood pressure compared with low-BMI trajectory. However, the mean increase of medium-BMI trajectory in SBP was higher than that in high-BMI trajectory, this conclusion was inconsistent with previous study,^[31]^ this study showed that BMI and SBP were positively correlated in adults. Although medium-BMI trajectory showed a downward trend, it was still at a relatively high level compared with low-BMI trajectory, its impact on the vascular system persisted even after the BMI decreased, and affected the increase of blood pressure in future years. Our results indicated that it had a positive effect on the increase of SBP. However, the influence of this trajectory on DBP became insignificant in Model 2, possibly because the impact of BMI trajectory on DBP might be greatly influenced by other metabolic factors.

Similarly, compared with low-BMI trajectory, the middle-BMI and high-BMI trajectory also had effects on future FBG. One study included 23,978 participants in Japan and compared BMI trajectory between undiagnosed and developing diabetes groups. The results showed that the FBG of high- (>30 kg/m^2^) and medium-BMI (>25 kg/m^2^) group whose did not develop diabetes were higher than that in low group and increased slowly over time during 9 years before diagnosis.^[32]^ A prospective study included 6223 residents (>55 years) from Rotterdam and 565 participants were confirmed with incident type 2 diabetes during a median follow-up period of 13.7 years. The result showed an increase in mean levels of FBG from 4.9 mmol/L to 9.4 mmol/L for the “progressive-overweight” (mean BMI = 28 kg/m^2^) and “persistently-high-BMI” (mean BMI = 35.4 kg/m^2^) groups.^[33]^ However, the baseline BMI in this study is much higher than our study, which were 28.0 kg/m^2^ and 35.4 kg/m^2^, respectively. Luo et al^[34]^ found that BMI trajectories in late middle age (50–60 years) were significantly associated with the risk of diabetes in older adults (≥60 years) in 11,441 participants. The results showed that compared to stable BMI trajectory, slowly-increasing-BMI trajectory had a 31% higher risk of developing diabetes, while rapidly increasing BMI trajectory had a 50% higher risk.

In subgroup analysis, we found that the association between BMI trajectory and future changes in blood pressure was stronger in men than that in women, while the association between BMI trajectory and FBG was slightly stronger in women than that in men. However, limited studies evaluated sex differences in the effects of BMI trajectory on blood pressure and FBG. One Chinese study reported that the association between increased BMI trajectories within the normal range and increased risk of hypertension was more pronounced in women.^[35]^ However, mean age was 45.6 ± 8.5 years and mean BMI were 21.9 ± 1.3 kg/m^2^ in above-mentioned study, which were obviously lower than our study. Another Iranian study reported that compare to “normal-weight” trajectory, the risk of developing high blood glucose of “overweight-to-late-obese” trajectory in women was higher than that in men.^[36]^ However, the study population was adolescent (12–20 years). Our study also showed that women and “older old” (≥70 years) with high-BMI trajectory had the same change in FBG. People aged 60 to 70 years and those aged over 70 years old exerted small differences in the association between BMI trajectory and FBG. There was a study showed that age, men and BMI are risk factors for increasing blood pressure.^[37]^

We found that the results of sensitivity analysis differed from the main analysis. This might be because a large number of participants with missing data and history of hypertension were excluded, and the reduction in sample size led to a weakened significance of some associations. After excluding diabetic patients, the association between high-BMI trajectory and FBG might weaken to be insignificant due to the reduction in FBG fluctuations in the sample.

## 5. Limitations

The advantage of our study included the availability of repeated measurements, and taken some traditional risk factors into consideration. However, there were some limitations in our study. First, changes in blood pressure and FBG are influenced by many other social factors, such as smoking, alcohol consumption, diet, and anti-hypertension or anti-diabetes drugs.^[38,39]^ However, all these information were deficient in our study. Second, the study population were from one community in Shanghai, thus the generalizability of the study was limited. Third, our study did not take into account medication using of participants during the follow up period. Finally, BMI remained stable within each BMI trajectory. We did not know if BMI reduce from high-level to low level or from the low level to high level, and how blood pressure and FBG changed. Further study included more participants is necessary to clarify above concerns and duplicate our results.

## 6. Conclusion

In this study, we identified 3 different trajectories and found that BMI trajectory was significantly associated with future increase in blood pressure and FBG in the Chinese aged population.

## Author contributions

**Conceptualization:** Yun Li.

**Formal analysis:** Xinyu Zhao, Ying Jiang.

**Methodology:** Yun Li, Renying Xu.

**Visualization:** Xinyu Zhao, Ying Jiang.

**Writing – original draft:** Xinyu Zhao.

**Writing – review & editing:** Yun Li, Renying Xu.

## Supplementary Material


