# Supplementary material for: The trajectory of body mass index and blood pressure and fasting blood glucose in Chinese aged population: Cohort study
Source: Medicine (Baltimore). 2026 Jan 9;105(2):e47159. doi: 10.1097/MD.0000000000047159 (PMC12795061; doi:10.1097/MD.0000000000047159)
Supplement: Supplementary file 1 [file medi-105-e47159-s001.docx]

Supplementary Figure 1. The recruitment of community based Chinese aged population

Abbreviation: SBP, systolic blood pressure; FBG, fasting blood glucose; HbA1c, glycosylated hemoglobin, type A1C; TG, total triglycerides.


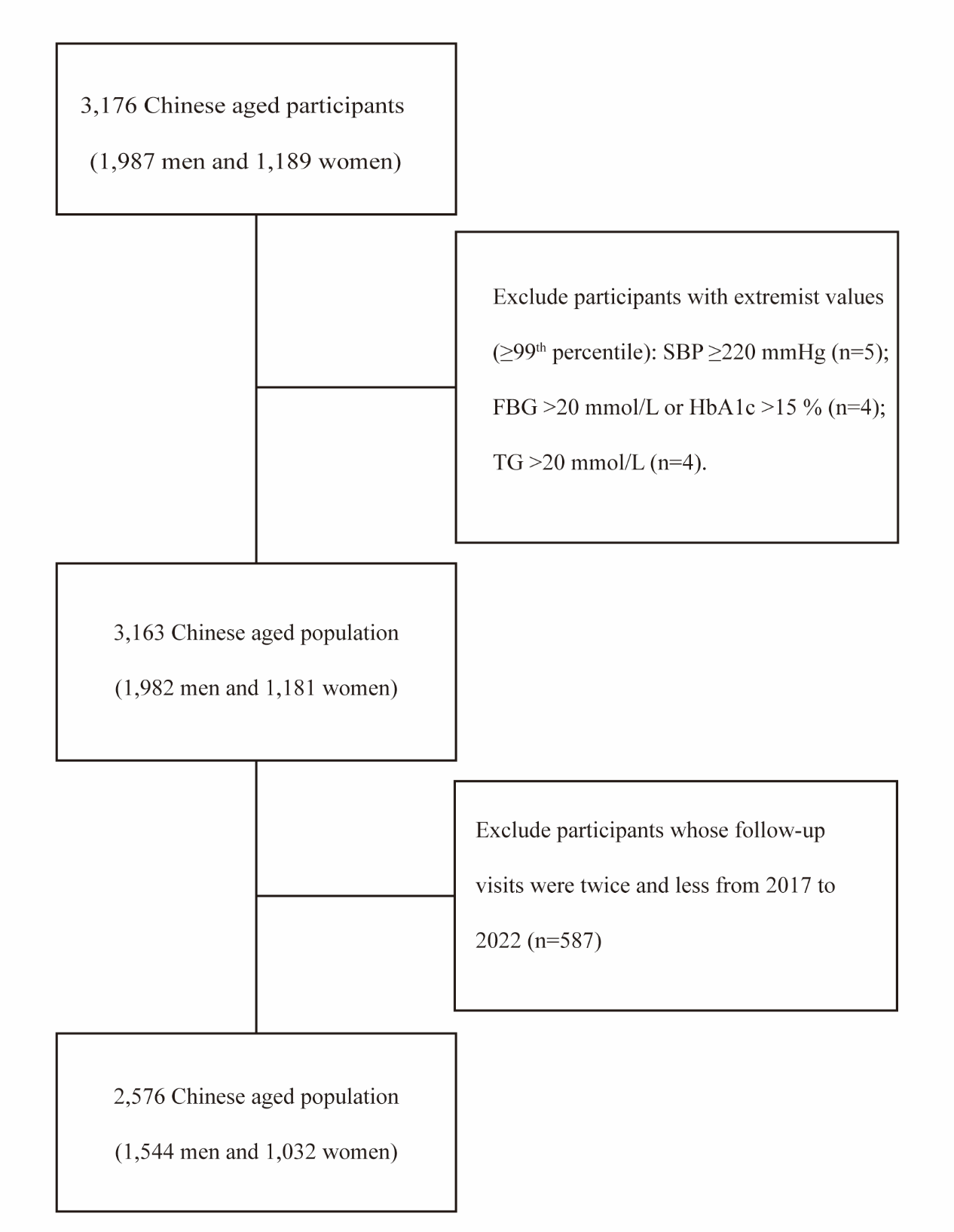


Supplementary Figure 2. The changes of systolic blood pressure in three BMI trajectories from 2014 to 2022.

Abbreviation: SBP, systolic blood pressure.

low-BMI medium-BMI high-BMI


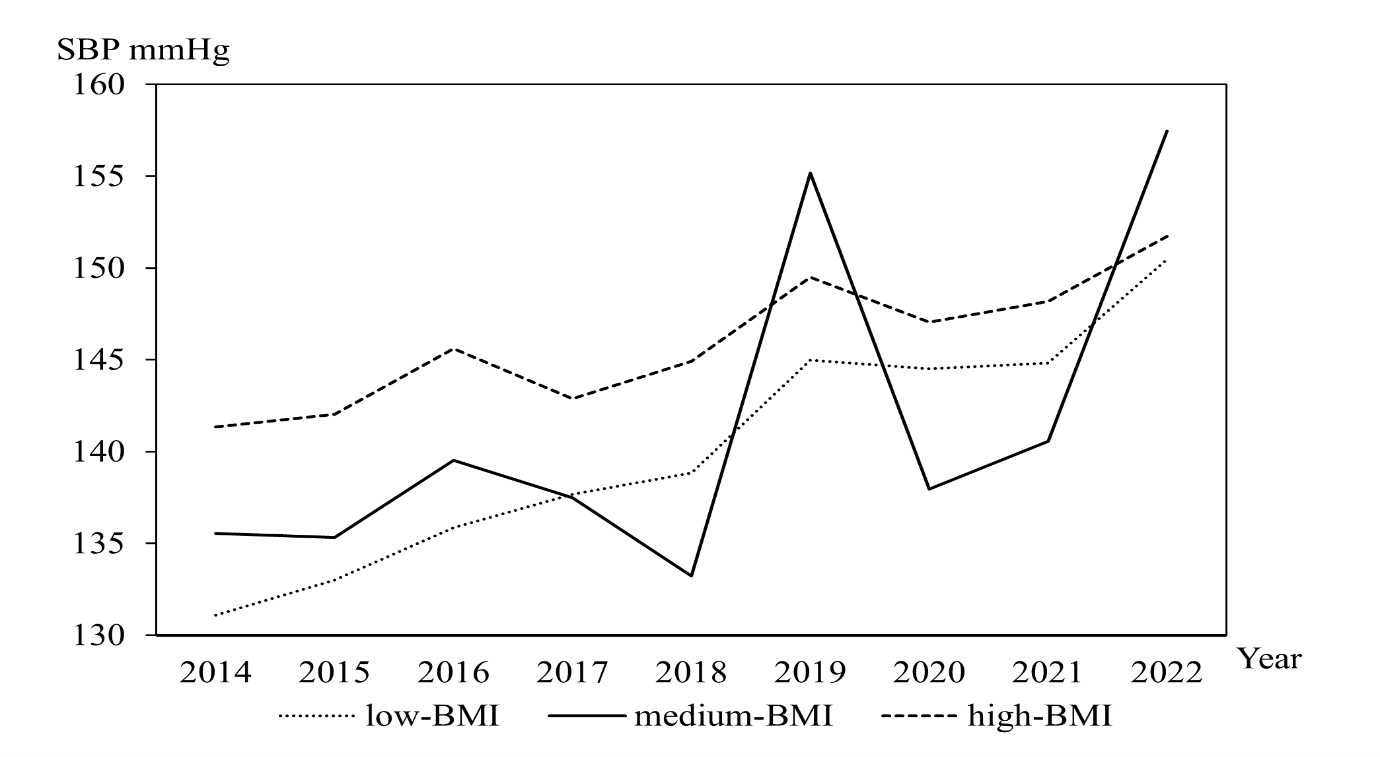


Supplementary Figure 3. The changes of diastolic blood pressure in three BMI trajectories from 2014 to 2022.

Abbreviation: DBP, diastolic blood pressure.

low-BMI medium-BMI high-BMI


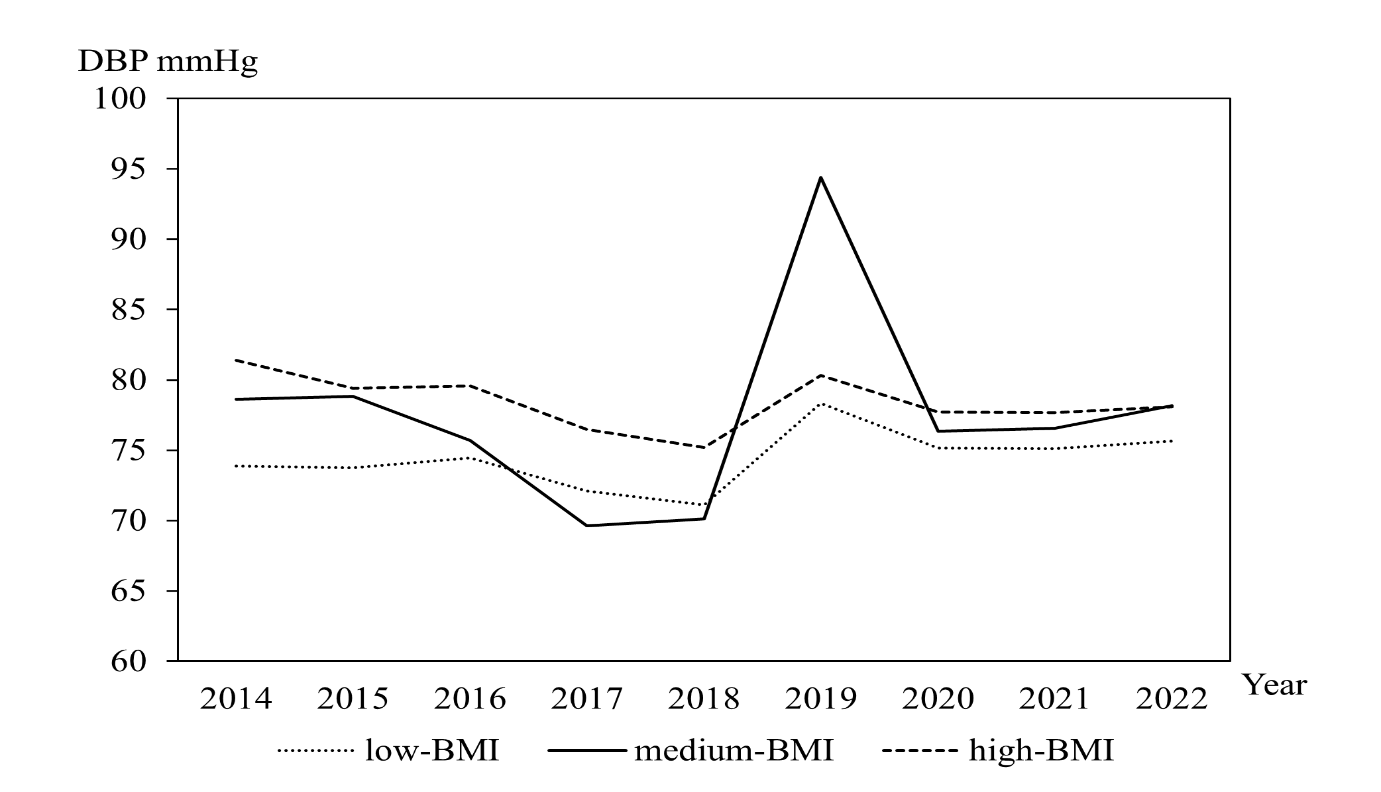


Supplementary Figure 4. The changes of fasting blood glucose in three BMI trajectories from 2014 to 2022.

Abbreviation: FBG, fasting blood glucose.

low-BMI medium-BMI high-BMI


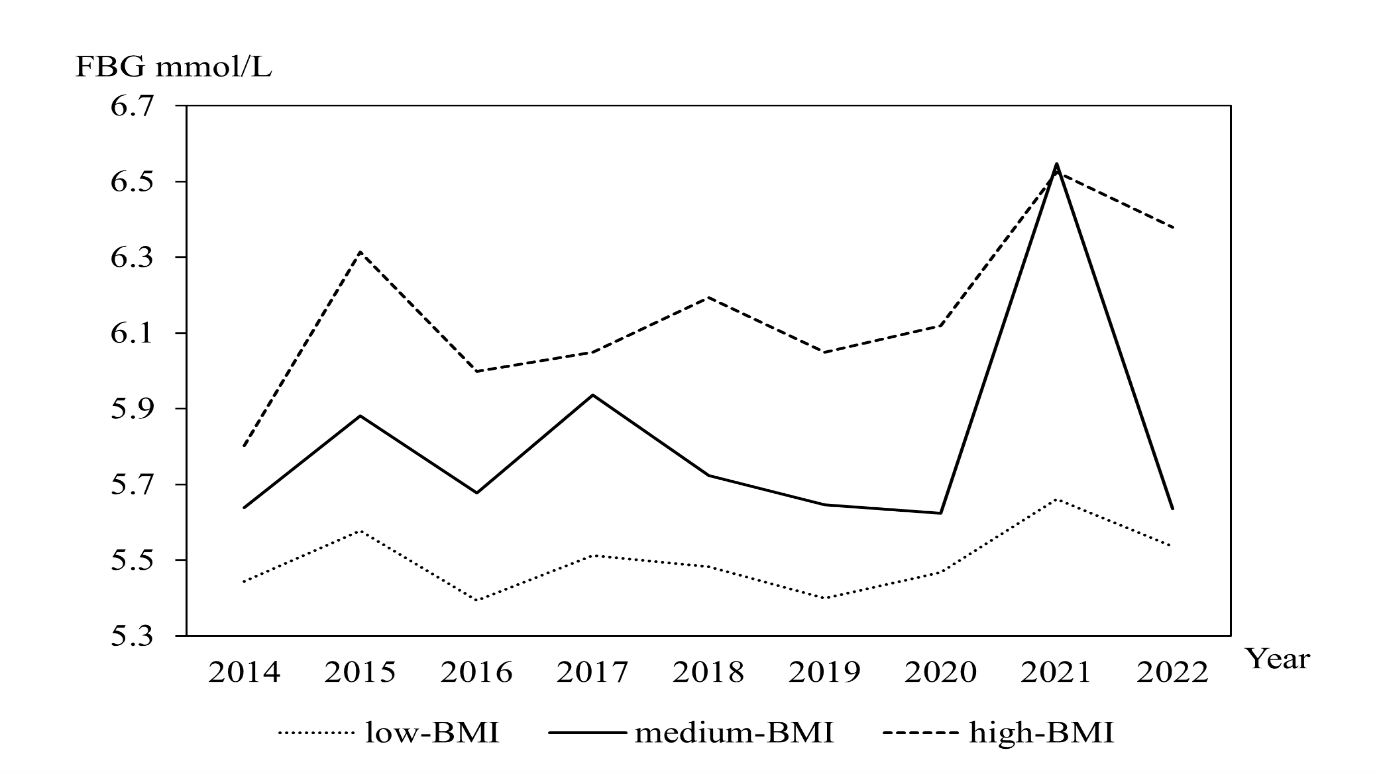


**Supplementary Table 1.** Basic characteristics between participants in and out of the study in 2016

|  | Out of the study (n=587) | In the study (n=2,576) | P value |
| --- | --- | --- | --- |
| Age, years | 70.2 ± 8.3 | 69.7 ± 6.7 | 0.16 |
| Height, cm | 165.0 ± 7.8 | 161.8 ± 8.1 | <0.001 |
| BW, kg | 67.0 ± 11.3 | 62.9 ± 11.0 | <0.001 |
| BMI, kg/m² | 24.6 ± 3.4 | 23.9 ± 3.0 | <0.001 |
| SBP, mmHg | 142.4 ± 17.7 | 139.8 ± 17.2 | 0.001 |
| DBP, mmHg | 78.3 ± 11.3 | 76.0 ± 10.3 | <0.001 |
| FBG, mmol/L | 5.8 ± 1.3 | 5.7 ± 1.1 | 0.046 |
| TC, mmol/L | 4.9 ± 1.0 | 5.3 ± 1.2 | <0.001 |
| TG, mmol/L | 1.3 (0.9, 1.9) | 1.6 (1.1, 1.8) | 0.022 |
| ALT, U/L | 18.0 (13.0, 23.0) | 16.0 (14.0. 22.0) | 0.008 |
| AST, U/L | 21.8 ± 8.1 | 20.4 ± 8.0 | <0.001 |
| EGFR, mL/min/1.73m² | 88.8 ± 20.5 | 81.8 ± 19.9 | <0.001 |
| HbA1c, % | 6.0 ± 0.9 | 6.0 ± 0.7 | 0.927 |

**Abbreviation**: BW, body weight; BMI, body mass index; SBP, systolic blood pressure; DBP, diastolic blood pressure; FBG, fasting blood glucose; TC, total cholesterol; TG, total triglycerides; ALT, alanine aminotransferase; AST, aspartate aminotransferase; EGFR, estimated glomerular filtration rate; HbA1c, glycosylated hemoglobin, type A1C.

**Supplementary Table 2.** The average BMI from 2014 to 2016 across different BMI trajectories in 2,576 Chinese aged participants

|  | Year | Low-BMI  (n=418) | Medium-BMI  (n=1,806) | High-BMI  (n=352) | P value |
| --- | --- | --- | --- | --- | --- |
| BMI, kg/m² | 2014 | 20.7 ± 1.5 | 24.8 ± 2.1 | 28.9 ± 2.2 | <0.001 |
|  | 2015 | 20.5 ± 1.6 | 25 ± 1.8 | 28.7 ± 2.0 | <0.001 |
|  | 2016 | 20.6 ± 1.6 | 23.7 ± 2.0 | 28.8 ± 2.2 | <0.001 |

**Note**: BMI, body mass index.

**Supplementary Table 3.** The mean difference and 95% CI for the association between BMI trajectory and future change in blood pressure and FBG using multiple imputed data

|  | Low-BMI (n=418) | Medium-BMI (n=1,806) | High-BMI  (n=352) | P value |
| --- | --- | --- | --- | --- |
| SBP |  |  |  |  |
| Model 1 | *0 (ref)* | 7.6 (5.1, 10.1) | 8.7 (5.5, 11.8) | <0.001 |
| Model 2 | *0 (ref)* | 4.5 (2.2, 6.8) | 3.1 (0.1, 6.2) | <0.001 |
| DBP |  |  |  |  |
| Model 1 | *0 (ref)* | 2.1 (0.6, 3.5) | 4.3 (2.5, 6.2) | <0.001 |
| Model 2 | *0 (ref)* | 1.0 (-0.3, 2.4) | 2.4 (0.5, 4.2) | <0.001 |
| FBG |  |  |  |  |
| Model 1 | *0 (ref)* | 0.4 (0.2, 0.6) | 0.7 (0.4, 0.9) | <0.001 |
| Model 2 | *0 (ref)* | 0.2 (0.0, 0.4) | 0.3 (0.1, 0.6) | <0.001 |

Note:

1.Abbreviation: SBP, systolic blood pressure; DBP, diastolic blood pressure; FBG, fasting blood glucose.

2.Model 1: Adjusted for age (years) and sex.

3.Model 2: Adjusted for variables in model 1 and further adjusted by systolic blood pressure (mmHg)，diastolic blood pressure (mmHg)，fasting blood glucose (mmol/L), total cholesterol (mmol/L), total triglycerides (mmol/L), alanine aminotransferase (U/L), aspartate aminotransferase (U/L) and estimated glomerular filtration rate (mL/min/1.73m²). If the analysis was performed in subgroup, the related variable was not included in the model (e.g., SBP was not included in the model when the analysis was performed with SBP).
